# Modelling growth of two *Listeria* monocytogenes strains, persistent and non-persistent: Effect of temperature

**DOI:** 10.1016/j.heliyon.2024.e40936

**Published:** 2024-12-07

**Authors:** Ľubomír Valík, Jana Minarovičová, Eva Kaclíková, Adriana Véghová, Tomáš Kuchta

**Affiliations:** a Department of Nutrition and Food Quality Assessment, Faculty of Chemical and Food Technology, Slovak University of Technology, Radlinského 9, 812 37, Bratislava, Slovakia; b Department of Microbiology, Molecular Biology and Biotechnology, Food Research Institute, National Agricultural and Food Centre, Priemyselná 4, 824 75, Bratislava, Slovakia

## Abstract

Better growth is a phenotypic trait that can contribute to persistence of *Listeria monocytogenes* in food processing environments. To test the hypothesis objectively, persistent and non-persistent strains were selected and grown in different media to gain reliable quantitative growth characteristics. In this study, the effect of temperature in the range from 6 °C to 43 °C on the planktonic growth of genotypically and phenotypically different strains LM9611-19 (LM-P, persistent) and LM120/5 (LM-S, sporadic - potentially non-persistent) in Tryptone Soy Broth (TSB) and in semi-synthetic cheese medium (SCM) was investigated. Two steps of growth modelling were applied to primary growth data and growth parameters using Baranyi and cardinal temperature models (CM), respectively. No statistically significant differences were found between the growth rates of the strains within the temperature range of 6 °C–37 °C in both media. However, the average growth rates were significantly higher (p < 0.05) for LM-P than for LM-S at 40 °C and 43 °C in both media. Regardless of whether calculated on *μ*_max_ or *λ* basis, in TSB or SCM, *T*_min_ for LM-P strain ranged from −1.2 to 0.7 °C with an average of 0.0 ± 0.9 °C (mean ± SD). Other averages of cardinal values were in TSB (a_w_ = 0.995; pH 7) *T*_opt_ = 37.8 ± 2.0 °C, *T*_max_ = 43.6 ± 0.5 °C and *μ*_opt_ = 1.27 ± 0.2 h^−1^. In SCM (a_w_ = 0.970, pH 7), the averages of *T*_opt_, *T*_max_, and *μ*_opt_ were 38.0 ± 1.2 °C, 45.2 ± 2.9 °C and 0.92 ± 0.04 h^−1^, respectively. Generally, the parameters of the CM model for the growth rate of sporadic strain in cheese medium were lower than for the persistent strain. This includes also *μ*_opt_, which reflects lower experimental growth rates in the range from *T*_opt_ to *T*_max_. However, based on the results found in the suboptimal temperature range, it seems that the growth rate did not play an important role in the persistency characteristics. It should be noted that the study was accompanied not only by low errors in model parameters but also by acceptable external validation indices for *μ*_max_ values in SCM. To consider the ComBase Predictor data (n = 8), the bias factors (*B*_f_) of 1.08 and 1.05 and accuracy factor (*A*_f_) of 1.09 and 1.07 were calculated for the LM-P strain and LM-S strain, respectively. The approach used in this study revealed different growth responses in the range of temperatures higher than *T*_opt_. It can be extended also for mild inactivation temperature range as similar differences in behaviour between persistent and non-persistent strains might also be expected.

## Highlights

• *T*_opt_ divided similarities and differences in the growth of persistent and sporadic strains under study.
• Similar growth parameters were found in a suboptimal temperature range.
• Statistically relevant differences were observed beyond the optimum temperature.
• Predictions for persistent strain using non-persistent strain data below *T*_opt_ were possible.

## 1 Introduction

*Listeria monocytogenes* is an important food-borne pathogen causing life-threatening disease in immunocompromised populations [1,2]. The main vehicle of infection is ready-to-eat food [3], which is often contaminated from the processing environment [[4], [5], [6]]. Except for a decrease in the number of cases to 1887 in 2020 (the COVID-19 pandemic period), the general trend for listeriosis did not show any significant increase or decrease in the last decade. However, the mortality rate in EU increased to 18.1 % in 2022, higher than in 2021 and 2020 (13.7 % and 13.0 %, respectively) [3].

*L. monocytogenes* is a Gram-positive, facultatively anaerobic, non-spore-forming, motile rod bacterium (in the range of 20–25 °C). Its cardinal growth parameters referring to the main intrinsic and extrinsic food environmental factors are well known (*T*_min_ = −1.5 °C, *T*_opt_ = 37 °C, *pH*_min_ = 4.39, *pH*_opt_ = 7.0, *pH*_max_ = 9.6, *a*_*w*__min_ = 0.92 *μ*_opt_ = 2.0 h^−1^) and are successfully used in growth predictions [7]. However, variability in growth should be expected and can be quantified considering experimental, biological, and strain differences [8].

Recently, the term microbial ‘persistence’ was defined as the ability of a given organism to be established in niches (or harbourage sites) within the food and feed processing environment for a long term, despite the regular application of cleaning and disinfection [9]. Long-term presence means months or years accompanied by growth or survival in specific places of the processing environment. Despite extensive research, the persistence mechanism of *L. monocytogenes* in the food processing environment remains a topic of interest [10,11]. The main candidate phenotypic traits responsible for persistence include increased biofilm formation [12,13], formation of mixed biofilms [14,15], higher resistance to sanitisers [16,17] and better growth in suboptimal conditions [18,19]. While the contribution of biofilm formation and resistance to sanitisers has been extensively discussed for *L. monocytogenes* persistence, this study aims to add information on the contribution of growth rate to this phenomenon.

Primary mathematical modelling enables the analysis of the behaviour of microorganisms involving spore germination, growth, inhibition, inactivation, or survival over time. It provides kinetic models with parameters that are, however, sensitive to changes in food environmental factors [20]. The mathematical relationships between the primary model parameters and the independent variables, such as intrinsic and extrinsic factors, such as *a*_*w*_, *pH*, temperature or modified atmosphere, are usually the subject of secondary modelling. Combining primary and secondary models, even more sophisticated tertiary models or expert systems can be created to predict the fate of microorganisms in specific foods with known environmental factors. This approach may help food microbiologists and manufacturers ensure the microbiological quality and safety of food throughout the food chain. For these reasons, mathematical modelling and predictive microbiology approach are integral parts of microbiological exposure assessment and, in combination with dose-response models, are also an inevitable part of risk assessment [21,22].

The present study focused on growth quantification and modelling using classical primary and secondary modelling to characterise the growth of a persistent and a sporadic *L. monocytogenes* strain. The main goal was to compare their growth responses in two media, Tryptic Soy Broth (TSB) and semi-synthetic cheese medium (SCM), and to assess the variability as well as the statistical significance of potential differences in their behaviour.

## 2 Materials and methods

### 2.1 Bacterial strains

*L. monocytogenes* LM9611-19 (LM-P) and *L. monocytogenes* LM120/5 (LM-S) were used as representatives of persistent and sporadic potentially non-persistent strains, respectively.

LM-P was isolated from ewes' milk stored in a cooling tank at a farm that produces raw milk cheese and identified by the State Veterinary and Food Institute, Dolný Kubín, Slovakia. It was classified as molecular serogroup IIa, clonal complex 14, sequence type 14 and was considered persistent based on <10 allelic differences in cgMLST (39 isolates during 18-month period) in our previous study [23,24]. Based on testing according to Stepanović et al. [25] and Di Ciccio et al. [26], the strain was classified as a moderate biofilm producer (data not shown). Upon testing substrate utilization with API Listeria (Biomérieux, Marcy l'Étoile, France), the strain gave typical results with a numerical profile 6510.

LM-S was isolated from the milking equipment filter in the same facility as LM-P and characterised in our laboratories. It was classified as molecular serogroup IIa, clonal complex 14, sequence type 91 and considered sporadic (single isolate for 18 months) in our previous study [23,24]. Based on testing according to Refs. [25,26], the LM-S strain was classified as a weak biofilm producer (data not shown). Upon substrate utilization testing with API Listeria, the strain gave atypical results of positive fermentation of D-xylose and D-tagatose, resulting in a numerical profile 6711.

### 2.2 Preparation of the basic bacterial suspension

For growth experiments, freeze-dried *L. monocytogenes* strains were revitalised by incubation in TSB (Merck, Darmstadt, Germany) at 37 °C for 24 h with mild shaking (1.7 Hz). The culture was streaked on TSA (Merck) and incubated at 37 °C for 48 h. Plates with cultures were stored at 5 °C with re-inoculation every 14 days for a maximum of 5 times. Cultures preserved in this way were used to prepare the basic suspension by picking a well-isolated colony into 10 ml of TSB, mixed with vortex, and incubated at 37 °C with mild shaking (1.7 Hz). After 18 h, microbial counts were determined by spreading 200 μl of decimal dilutions in peptone saline (containing 8.5 g NaCl (Mikrochem, Pezinok, Slovakia) and 1 g peptone (Lab M, Heywood, United Kingdom) per 1000 ml) on the surface of TSA plates in duplicate. Counts were calculated and expressed according to ISO 4833–2 [27]. The numbers determined for the basic suspension were used to adjust the inoculum so that, after adding 1 ml–300 ml of growth medium, the concentration at the beginning of each experiment was within the range of 10^2^–10^3^ CFU/ml.

### 2.3 Semi-synthetic cheese medium

The semi-synthetic cheese medium (SCM) was prepared according to Kagkli et al. [28] and Schrama et al. [29] to imitate the nutritive potential of cheese. The following components were dissolved in 275 ml deionised water: 0.06 g CaCl_2_·6H_2_O (Sigma-Aldrich, St. Louis, USA), 0.30 g yeast extract (HiMedia, Mumbai, India), 0.31 g MgSO_4_·6H2O (Lachema, Brno, Czech Republic), 1.8 g L-methionine (Merck, Darmstadt, Germany), 2.04 g of KH_2_PO_4_ (Centralchem, Bratislava, Slovakia), 3 g NaCl (Centralchem), 4.5 g casein (bioreagent for cell culture grade, Sigma-Aldrich) and 11.4 ml sodium DL-lactate (Sigma- Aldrich). The lactose stock solution was prepared by steam sterilisation at a temperature of up to 100 °C for 20 min. After cooling, 25 ml of the solution was added aseptically to the cheese medium base which finally contained 8.4 g of lactose (Slavus, Bratislava, Slovak Republic) in 300 ml. Using a 10 % NaOH solution (Mikrochem) or lactic acid (Lachema), the pH value of the complete medium was adjusted to pH 7.2 before sterilisation to obtain pH 7.0 ± 0.1 after sterilisation. The *a*_*w*_ value of the medium was 0.974 ± 0.003.

### 2.4 Quantification of L. monocytogenes and cultivation experiments

A volume of 1 ml of the appropriate dilution of the basic bacterial suspension was aseptically added to a flask with 300 ml of TSB or SCM and thoroughly mixed. Inoculated growth media were incubated at temperatures of 6 °C, 10 °C, 16 °C, 22 °C, 25 °C, 30 °C, 37 °C, 40 °C and 43 °C. For quantification, 1 ml of the culture from each flask was taken at predetermined time intervals over a period until the bacterial population reached a stationary phase or for up to 14 h. Subsequently, 200 μl of certain set dilutions were plated on the TSA surface in duplicate. The agar plates were incubated at 37 °C for 24–48 h.

Duplicate growth curves were constructed within each experiment in TSB at each temperature, except for 6 °C, 30 °C and 43 °C, at which two independent experiments were carried out. In SCM, two independent experiments were carried out in duplicates at each temperature.

### 2.5 Evaluation of variability

A) Inspired by Aryani et al. [8,30], who defined the experimental, biological, and strain variabilities for growth and inactivation rates of 20 strains of *L. monocytogenes* in detail, indices such as MSE and RMSE were estimated in this study. Indices were calculated for the growth parameters of LM-P and LM-S strains at each temperature. According to the mentioned authors, experimental variability was defined as the difference between duplicate experiments conducted in parallel at the same time on the same experimental day. Biological variability was defined as the difference between independently reproduced experiments of the same strain performed on different experimental days from new pre-cultures and newly prepared media, and strain variability means the difference between strains of the same species.
B) Alternatively, experimental RMSE values were also calculated from primary growth curve data (experiments with two replicates). Then, biological RMSE resulted from the combined data of 2 experiments, each with 2 replicates (4 replicates altogether), and analogously, the strain RMSE was calculated from all combined growth data for both strains in each medium used. These data sets involved data from 4 experiments (8 replicates; Table 1 and Table 2 for 6 °C and 30 °C, respectively). The mentioned temperatures were chosen because they generally represent low and optimal growth temperature ranges, and also because the highest number of replicates was available at these temperatures. This approach can provide an additional perspective to the evaluation of experimental, biological, and strain variabilities.

**Table 1:** Growth data of *L. monocytogenes* strains in TSB and SCM at 6 °C.

Strain	*n*_rep_/*n*_exp_	Medium	Model parameters					Statistical indices			*n*
			*λ* (h)	rate	log *N*_0_	log *N*_end_	**RMSE**	**RMSE**_**ave**_	Variability	R^2^	
				(log CFU/h)	(log CFU/mL)						
LM-P	2/1	TSB	45.0	0.022	3.58	9.24	0.063	**0.072**	experimental	0.999	15
			42.6	0.022	3.55	9.43	0.084			0.998	15
			70.0	0.022	3.22	8.97	0.072			0.998	12
			67.0	0.022	3.19	9.00	0.069			0.999	12
LM-S			78.7	0.020	3.48	9.71	0.109	**0.080**		0.997	12
			59.4	0.022	3.31	9.49	0.095			0.998	13
			87.7	0.022	3.14	8.98	0.042			0.999	12
			89.6	0.021	3.05	9.15	0.075			0.999	13
LM-P	2/1	SCM	98.9	0.020	3.18	7.65	0.110	**0.121**	experimental	0.996	11
			107.9	0.021	3.23	7.52	0.211^a^			0.987	11
			103.9	0.021	3.36	7.63	0.072			0.998	13
			108.8	0.023	3.27	7.58	0.091			0.997	13
LM-S			116.3	0.025	2.53	7.49	0.361^a^	**0.264**		0.970	11
			116.0	0.021	2.52	7.63	0.333^a^			0.971	11
			127.5	0.024	2.94	7.30	0.264^a^			0.979	16
			95.9	0.020	2.99	7.55	0.100			0.997	17
LM-P	4/2	TSB	42.3	0.021	3.33	9.21	0.388	**0.289**	biological	0.964	54
LM-S			78.9	0.021	3.28	9.45	0.371			0.968	50
LM-P		SCM	104.0	0.021	3.28	7.60	0.133			0.994	48
LM-S			105.4	0.020	2.89	7.55	0.266			0.978	55
**LM-P + LM-S**	**8/4**	TSB	**51.9**	**0.020**	**3.25**	**9.35**	**0.487**	**0.394**	strain	0.945	104
**LM-P + LM-S**	**8/4**	SCM	**105.0**	**0.020**	**3.10**	**7.56**	**0.301**			0.971	103

a Declination of the numbers observed during the lag phase. *n*_*r*ep_ - number of replicates within the experiment, *n*_exp_ - number of experiments.

**Table 2:** Growth data of *L. monocytogenes* strains in TSB and SCM at 30 °C.

Strain	n_rep_/n_exp_	Medium	Model parameters					Statistical indices			n
			*λ* (h)	rate	log *N*_0_	log *N*_end_	**RMSE**	**RMSE**_**ave**_	Variability	R^2^	
				(log CFU/mL/h)	(log CFU/mL)						
LM-P	2/1	TSB	2.6	0.469	3.58	9.45	0.106	**0.069**	experimental	0.998	16
			2.5	0.464	3.55	9.43	0.075			0.999	16
			2.0	0.452	3.63	9.34	0.041			0.999	15
			1.7	0.446	3.57	9.38	0.054			0.999	16
LM-S			3.3	0.492	3.07	9.56	0.113	**0.084**		0.998	15
			3.2	0.493	3.06	9.52	0.107			0.998	15
			2.5	0.464	3.16	9.38	0.048			0.999	16
			2.5	0.475	3.17	9.45	0.068			0.999	16
LM-P	2/1	SCM	3.6	0.383	3.80	7.93	0.107	**0.085**	experimental	0.996	14
			4.1	0.439	3.77	7.82	0.103			0.997	12
			4.0	0.363	3.75	8.03	0.081			0.998	18
			3.4	0.344	3.73	7.97	0.049			0.999	18
LM-S			4.4	0.315	3.27	8.01	0.130	**0.085**		0.995	18
			3.5	0.308	3.21	8.00	0.089			0.998	18
			3.2	0.351	2.68	7.94	0.057			0.999	21
			2.8	0.331	2.68	8.10	0.066			0.999	21
LM-P	4/2	TSB	2.3	0.475	3.49	9.25	0.181	**0.157**	biological	0.994	63
LM-S			2.8	0.476	3.10	9.48	0.150			0.996	62
LM-P		SCM	3.7	0.372	3.76	7.93	0.131			0.994	62
LM-S			3.5	0.326	2.96	8.00	0.167			0.992	78
**LM-P + LM-S**	**8/4**	**TSB**	**2.3**	**0.475**	**3.49**	**9.25**	**0.181**	**0.312**	strain	**0.985**	**125**
**LM-P + LM-S**	**8/4**	**SCM**	**3.5**	**0.341**	**3.36**	**7.79**	**0.444**			**0.937**	**140**

*n*_*r*ep_ - number of replicates within the experiment, *n*_exp_ = number of experiments.

### 2.6 Primary and secondary modelling

The density of the cultures of both *L. monocytogenes* strains was evaluated as a function of time in the two growth media according to Baranyi's D-model [31] using the Excel tool DMFit v. 3.5 (Institute of Food Research, Norwich, United Kingdom). To evaluate the effect of temperature on specific growth rate and lag phase (secondary modelling), non-linear regression using Excel Solver (Microsoft, Redmont, USA) was applied.

Baranyi's model is described by the following equations:

(1) $$y\left(t\right)=y_{0}+\mu_{\max}\cdot A\left(t\right)-\frac{1}{m}\cdot \ln\left(1+\frac{e^{m\cdot \mu_{\max}\cdot A\left(t\right)}-1}{e^{m\cdot \left(y_{\max}-y_{0}\right)}}\right)$$

(2) $$A\left(t\right)=t+\frac{\ln\left(e^{-\mu_{\max}\cdot t}+e^{-h_{0}}-e^{-\mu_{\max}\cdot t-h_{0}}\right)}{\mu_{\max}}$$

where *y*(t) = ln *N*(t), *y*_0_ = ln *N*_0_, *y*_max_ = ln *N*_max_, m is the parameter of the curvature of the growth line when the growth of microorganisms slows down from the exponential phase to the stationary phase. *A*(t) is an integral form of the adjustment function *α*(*t*) whose value increases with increasing time, *t* is time and *h*_0_ is a dimensionless factor that quantifies the initial physiological state of cells and is related to lag phase (*λ*) by the equation *h*_0_ = *μ*·*λ* [31].

Using log_10_ *N* in the DMFit tool, the main outputs of primary modelling were the growth rate *k*_max_ (log CFU/mL/h) and the duration of lag phase *λ* (h). Before secondary modelling, *k*_max_ was converted to *μ*_max_ (*h*^−1^) according to the equation $\mu_{\max}=k_{\max}\cdot \ln\left(10\right)$. To describe the effects of temperature on the growth of *L. monocytogenes* strains in TSB and SCM at *a*_*w*_ values of 0.995 and 0.974, respectively, the square root Cardinal model (sqrtCM) of Rosso et al. [32] was used in the following form:

(3) $$\sqrt{\mu_{\max}}=\sqrt{\mu_{opt}\cdot CM\left(T\right)}$$

(4) 1λ=1λopt⋅CM(T)

where

(5) $$CM\left(T\right)=\frac{\left(T-T_{\max}\right)\cdot {\left(T-T_{\min}\right)}^{2}}{\left(T_{opt}-T_{\min}\right)\cdot \left[\left(T_{opt}-T_{\min}\right)\cdot \left(T-T_{opt}\right)-\left(T_{opt}-T_{\max}\right)\cdot \left(T_{opt}+T_{\min}-2T\right)\right]}$$

In this work, a square root transformation of the whole cardinal model was used for both growth parameters *μ*_max_ and reciprocal *λ*. The secondary model includes five parameters with biological meaning, namely, *μ*_opt_, *λ*, *T*_min_ (theoretical minimum temperature), *T*_opt_ (optimum temperature) and *T*_max_ (maximum temperature above which growth is not likely). Except for normalising residuals, the modification allowed the estimation of parameters *μ*_opt_ and 1/*λ*_opt_ in the original non-squared form, which is more practical particularly for *μ*_opt_ (Eqs. (3), (4))).

### 2.7 Statistical analysis and model validation

Specific growth rates of the two strains at 6–37 °C were used for internal cross-validation, in which a set of *μ*_max_ _calc_ values for LM-P was validated with LM-S *μ*_max_ _exp_ values and vice versa. Due to statistically significant differences in *μ*_max_ values, the data at 40 °C and 43 °C were excluded from this validation.

External validation of secondary rate models for both strains in cheese medium was performed using growth rates acquired from the ComBase Predictor (CBP) at *a*_*w*_ 0.974, *pH* 7. For CMs in TSB, the experimental *μ*_max_ values estimated by Bajard et al. [33] in Mueller-Hinton broth (MHB) were used for validation. Data are collected in Suppl. Table S1.

To evaluate the accuracy of fitting, the coefficient of determination (*R*^*2*^) and the root mean square error (*RMSE*) were calculated:

(6) $$R^{2}=\sum_{i=1}^{n}\frac{{\left(y_{i}^{obs}-y_{i}^{cal}\right)}^{2}}{{\left(y_{i}^{obs}-{\bar{y}}_{i}^{cal}\right)}^{2}}$$

(7) $$RMSE=\sqrt{\frac{{\left(y_{i}^{obs}-y_{i}^{cal}\right)}^{2}}{n-p}}$$

where $y_{i}^{obs}$, $y_{i}^{cal}$ and ${\bar{y}}_{i}^{cal}$ are the observed (experimental), calculated (predicted) and averaged *μ*_max_ or *λ* data, respectively; *n* is the number of observations; and *p* is the number of model parameters [34].

The CMs for growth rate were externally validated using independent data excerpted from the Combase database at aw of 0.974 and pH 7 as well as the data from Ref. [33]. The following equations were used to calculate validation indices bias and accuracy factors [7], [32]:

(8) $$B_{f}=10^{\frac{\sum_{i=1}^{n}\left(\log\;{\mu_{max_{i}}}^{obs}-\log\;{\mu_{max_{i}}}^{pred}\right)}{n}}$$

(9) $$A_{f}=10^{\left(\sum_{i=1}^{n}\left|\log\;{\mu_{max_{i}}}^{pred}-\log\;{\mu_{max_{i}}}^{obs}\right|/n\right)}$$

According to te Giffel and Zwietering [7], the bias factor (*B*_f_) provides information on the distribution of the observed values towards the identity line between the observed (experimental) and predicted (calculated) *μ*_max_ values. In this case, a value of *B*_f_ lower than 1 indicates a ‘fail-safe’ prediction and a value of *B*_f_ greater than 1 means a ‘fail-dangerous’ model. The accuracy factor (*A*_f_) describes how close are the predicted *μ*_max_ to *μ*_obs_ values.

The statistical differences between *μ*_max_ values of the two *L. monocytogenes* strains at each temperature were statistically evaluated using the Analyse-it Method Validation package ver. 6.15 (Analyse-it Software, Leeds, United Kingdom). Data were treated using Student's t-test followed by Tukey's test with the least significant difference of 95 %.

## 3 Results and discussion

### 3.1 Growth of L. monocytogenes strains

In this work, 63 independent cultivation experiments were carried out with duplicate trials for the two strains, persistent LM-P and sporadic – potentially non-persistent LM-S, in TSB and SCM. TSB represented a theoretically optimal culture medium (*a*_*w*_ of 0.995, pH 7.0), while the semi-synthetic cheese medium simulated conditions that more closely resembled the internal environment of cheese (*a*_*w*_ of 0.947 ± 0.003, pH 7.0). The nine cultivation temperatures used (6–43 °C) covered almost the entire range of *L. monocytogenes* growth. In total, more than 120 growth curves were provided with growth parameters and statistical indices. Experimental, biological and strain variability was evaluated according to Aryani et al. [8] using specific growth rates and were also based on the RMSE values of the growth curves from individual experiments or combined data from 2 to 4 experiments (4–8 replicates) performed at different times.

### 3.2 Evaluation of variability

To quantify the internal experimental, biological and strain variability of *μ*_max_ according to Aryani et al. (2015a), we used *μ*_max_ data recalculated from growth rates of all individual curves carried out in SCM at each temperature. In this way, we could evaluate the MSE values and compare RMSE values for our persistent and sporadic strains with those of 20 strains by Aryani et al. [8,30]. The variances of *μ*_max_ for our strains were independent of temperature. The following RMSE_exp_, RMSE_biol_, and RMSE_str_ of 0.016 h^−1^, 0.022 h^−1^, and 0.017 h^−1^ were calculated, respectively, for both strains LM-P and LM-S in this study. Aryani et al. [8] observed almost identical variability and the same trend between the values as we did in the current study, as RMSE_biol_ was the highest, followed by RMSE_str_ and RMSE_exp_. However, in the second case the RMSE values of growth curves, which are log_10_*N*-based and express the goodness of fit for the primary growth model, can also provide an opportunity to evaluate experimental, biological and strain variability. The RMSE average values (RMSE_ave_) may come not only from individual trial duplicates conducted at the same time (experimental variability) or from experiments with duplicates performed at different times (biological variability) but also, in our case, from data combined from experiments with LM-P and LM-S (strain variability).

Together with other growth parameters, the RMSE values are presented in Table 1, Table 2 for temperatures of 6 °C and 30 °C, respectively. The experimental RMSE values fluctuated between 0.063 and 0.130 log CFU/mL for both strains, temperatures, and media with almost the same mean RMSE_exp_ of 0.082 and 0.081 for 6 °C and 30 °C, respectively. Similarly, the RMSE_ave_ values representing biological variability (2 experiments performed at different times containing data from 4 individual replicates) were 0.289 and 0.157 log CFU/mL for 6 °C and 30 °C, respectively. Finally, the highest average RMSE values of 0.394 and 0.312 log CFU/mL described the strain variability in both media (TSB and SCM) at 6 °C and 30 °C, respectively.

**Fig. 1:**
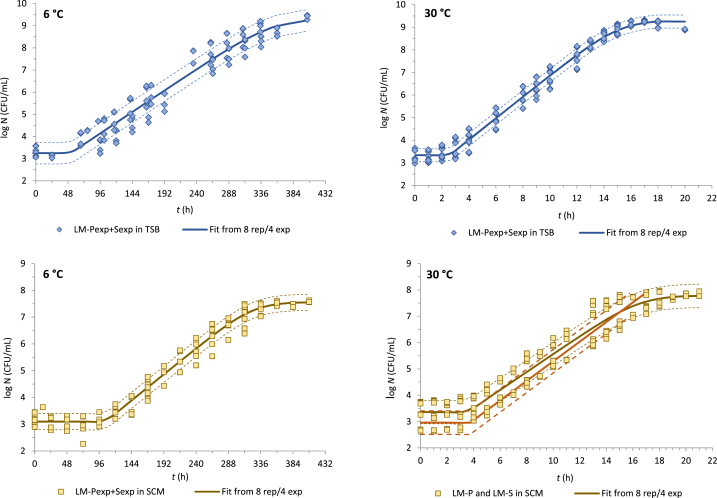
Growth curves of *L. monocytogenes* in TSB (blue) and SCM (yellow) at 6 °C and 30 °C. Merged data for LM-P and LM-S strains (LM-P_exp_ + S_exp_) come from 4 experiments and 8 replicates in total. Dashed lines represent fitted values ± standard error (SE) of the fit and the solid red line in the right button figure represents the recalculated growth curve using equalized *N*_0_ of 2.96 log CFU/mL.

In addition, the growth curves fitted with the Baranyi D-model using experimental data for both strains (LM-P_exp_ + LM-S_exp_; 8 replicates) and representing strain variability in each medium are shown in Fig. 1 (TSB in blue; SCM in yellow). We suppose that similar values could be found in the suboptimal temperature range. However, it could not be properly demonstrated as only one experiment with two replicates was performed in TSB at other temperatures within the study.

The comparison of variability based either on specific growth rates or fitting errors of growth curves needs to use relative indices. To do so, the ratios between the average RMSE values and *μ*-values were calculated for replicates, experiments, and combined strain data. Similarly, for relative indices based on growth curves, the ratio of RMSE and growth increases (log*N*_end_ - log*N*_0_) averages was used. The experimental, biological and strain variabilities based on *μ* data were represented with relative errors of 5.2 %, 4.0 % and 8.3 %, respectively. On the other hand, the relative indices based on the log*N* data showed slightly lower values of 1.6 %, 2.9 % and 6.1 %, respectively. It seems that both methods of evaluation provided acceptable outputs and, in this case, also certain relations between them could be defined. Except for one case, the relative indices doubled from experimental to biological and from biological to strain variability.

### 3.3 Effect of temperature on growth parameters of *L. monocytogenes* strains in TSB and SCM

**Fig. 2:**
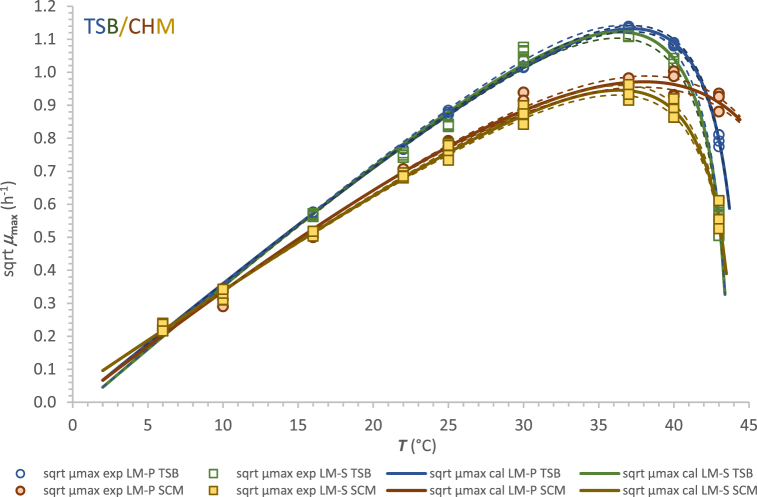
Modelling the effect of temperature on sqrt *μ*_max_ of LM-P and LM-S strains in Tryptone Soy Broth (TSB) and semi-synthetic cheese medium (SCM).

**Fig. 3:**
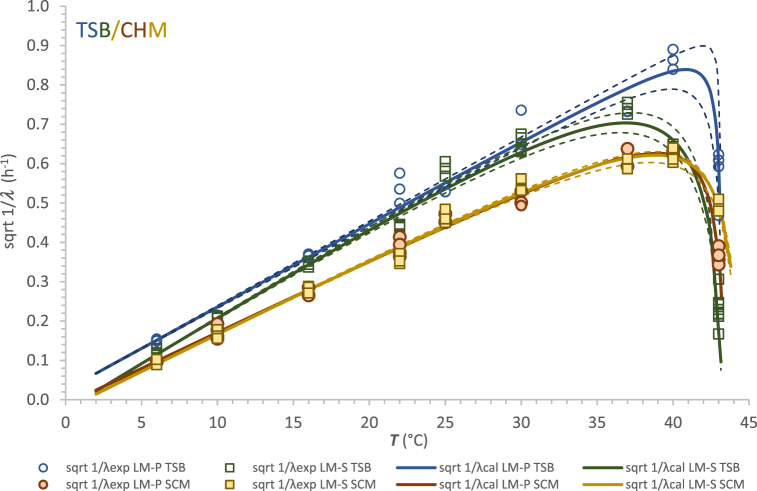
Modelling the effect of temperature on sqrt 1/*λ* of LM-P and LM-S strains in Tryptone Soy Broth (TSB) and semi-synthetic cheese medium (SCM).

The cardinal temperature model with inflection (CM) is one of the most widely used secondary models that reliably describes the effect of temperature in the full range of growth on microbial growth parameters. The model is often preferred as it provides four parameters that have biological interpretation. In contrast, the extended square root model contains two regression coefficients and parameters *T*_min_ and *T*_max_, which are only theoretical minimum and maximum temperatures for growth and may differ significantly from the actual cardinal temperatures [35]. Graphic presentations of the dependences of sqrt *μ*_max_ and sqrt 1/*λ* on the temperature for LM-P and LM-S in TSB (a_w_ 0.995) and SCM (a_w_ 0.974) are shown in Fig. 2, Fig. 3, respectively. The dashed lines represent the calculated data using standard errors of model parameters, which are summarised in Table 3.

**Table 3:** Parameters of the square root CM model and statistical indices of fit for LM-P and LM-S strains in TSB and SCM.

Parameters	**CM sqrt 1/λ LM** (TSB)				**CM sqrt *μ***_**max**_**LM** (TSB)				**CM sqrt 1/λ LM** (SCM)				**CM sqrt *μ***_**max**_**LM** (SCM)			
	LM-P	SE	LM-S	SE	LM-P	SE	LM-S	SE	LM-P	SE	LM-S	SE	LM-P	SE	LM-S	SE
*T*_min_ (°C)	−1.151	0.012	1.076	0.008	0.197	0.001	0.821	0.003	0.675	0.003	1.252	0.007	0.086	0.001	−1.115	0.005
*T*_opt_ (°C)	40.820	0.134	36.895	0.133	37.203	0.030	36.376	0.066	38.923	0.105	38.811	0.103	38.026	0.091	36.367	0.063
*T*_max_ (°C)	43.223	0.045	43.180	0.015	44.303	0.021	43.580	0.018	43.356	0.017	44.233	0.097	49.518	0.466	43.890	0.029
1/*λ*_opt_ (h^−1^)	0.704	0.024	0.495	0.016	–		**-**		0.392	0.009	0.387	0.010	–		–	
*μ*_opt_ (h^−1^)	–		–		1.282	0.009	1.259	0.020	–		–		0.944	0.018	0.894	0.013
**RMSE**	0.040		0.038		0.011		0.030		0.018		0.021		0.026		0.022	
**R**^**2**^	**0.971**		**0.970**		**0.999**		**0.991**		**0.989**		**0.987**		**0.991**		**0.992**	

The solid lines representing the sqrtCM model with sqrt *μ*_max_ for both strains in both media agreed with the observed *μ*_max_ in the suboptimal temperature range. Differences were found in the region beyond T_opt_ (which is approximately 37 °C; Fig. 2). The *μ*_max exp_ values for LM-P and LM-S strains in TSB and SCM did not show significant differences in the suboptimal temperature range (6 °C–30 °C) and at 37 °C in both media. At the 5 % significance level, this was confirmed by ANOVA, Student's t-test, and Tukey's test at each temperature. However, in the region between optimal and maximum temperature, which contained two experimental temperatures (40 °C and 43 °C), the differences between *μ*_max exp_ values from repeated trials were statistically confirmed for both strains and both media (*p* = 0.0229 and 0.0145 at 40 °C and *p* < 0.0001 and 0.0053 at 43 °C in TSB and SCM, respectively). Interestingly, the values of sqrt *μ*_max_ were higher for LM-P than for LM-S at the three highest incubation temperatures, but they were statistically significant only at the last two, 40 °C and 43 °C. Furthermore, both cardinal parameters *T*_opt_ and *T*_max_ determined by the sqrtCM model for sqrt *μ*_max_ were also higher for LM-P than for LM-S in both media (Table 3). In terms of growth rate, Magalhães et al. [19] found that persistent and non-persistent strains showed different responses to NaCl and acidic conditions (pH 5). Average rates were significantly higher (p < 0.05) for persistent than for non-persistent isolates when grown at 22 °C, 2.5 %, 4 %, 8 % NaCl, and at pH 5. In our case, the situation was different as significantly higher growth rates were observed for the persistent strain in both media only at temperatures higher than 37 °C (at 40 °C and 43 °C).

A similar trend could be observed in Fig. 3, which is the output of the sqrtCM model application to the lag phase. In this case, higher values of sqrt 1/*λ,* which means a shorter lag phase, could be seen more clearly in the region of temperatures beyond *T*_opt_. Likewise, the *T*_opt_ values resulting from secondary modelling of sqrt 1/*λ* against temperature were higher in the case of LM-P in both media (Table 3).

Magalhães et al. [19] estimated similar lag times for persistent and non-persistent isolates when grown at 37 °C, 22 °C and 4 °C but significantly shorter (p < 0.05) for persistent strains grown at 2.5 %, 4 % and 8 % NaCl, and at pH 5. In our case, significantly shorter (p < 0.05) lag times were observed for persistent strain (in both media) only at temperatures 40 °C. and 43 °C. However, the estimated *T*_max_ values from the lag phase modelling were within the range of 43.2–44.2 °C for both strains and the media.

The sqrtCM model fitted well all the data obtained from our experiments, as documented not only by the standard error of the parameters but also by the values of other statistical parameters such as RMSE and coefficient of determination *R*^2^ (Table 3). Associated with the minimal errors of estimation, all cardinal parameters for *L. monocytogenes* strains in this study were consistent with those mentioned by Bajard et al. [33], te Giffel and Zwietering [7] and Jia et al. [36]. Regardless of whether calculated on *μ*_max_ or *λ* basis, in TSB or SCM, for a persistent or non-persistent strain, the most discussed cardinal parameter *T*_min_, here with estimation errors from 0.0003 °C to 0.012 °C, ranged from −1.2 °C to 1.3 °C. For LM-P strain, the range of *T*_min_ values determined with the CM model in the two media was −1.2 to 0.7 °C with an average of 0.0 ± 0.8 °C (mean ± SD; n = 4). As in Table 3, strain LM-S showed a slightly higher average *T*_min_ of 0.5 ± 1.1 °C. A similar estimated minimum growth temperature of 0.6 ± 0.2 °C was reported for *L. monocytogenes* in pasteurised milk by Jia et al. [36].

### 3.4 Model validation

**Fig. 4:**
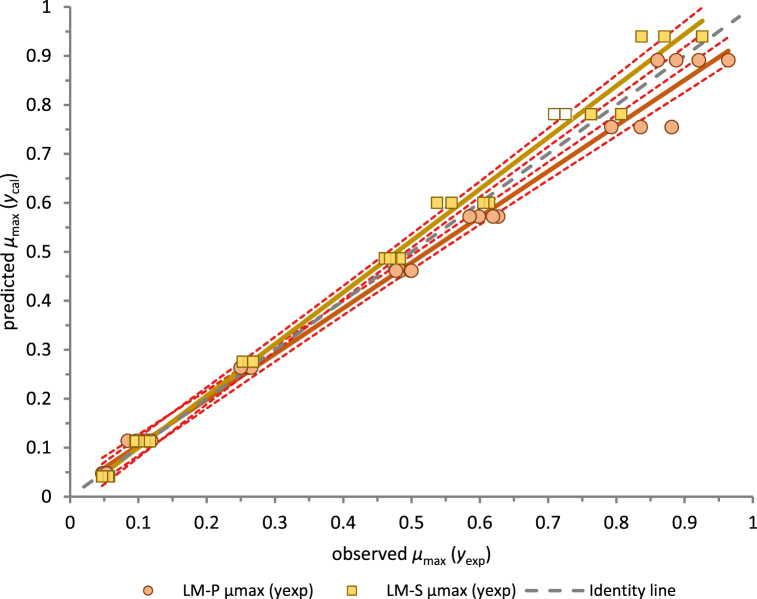
Comparison of the observed and predicted values at internal cross-validation with LM-P and LM-S in SCM.

Even with sound statistical indices of the sqrtCM and model parameters shown in Table 3, the results should be validated using independent data. In this work, growth data for two strains with different genetic and physiological characteristics offered an option of internal cross-validation, within which the sqrtCM model for LM-P could be validated with *μ*_max exp_ values of LM-S and vice versa. As statistically significant differences were found between the *μ*_max_ model and experimental data at 40 °C and 43 °C for the two strains, these data were excluded from internal cross-validation. Fig. 4 shows the comparison of two sets of predicted and observed *μ*_max_ values (recalculated from sqrtCM) in SCM at a_w_ of 0.974 for the suboptimal temperature range (6 °C–37 °C).

Table 4 provides accompanying linear regression data for the predicted and observed *μ*_max_ values with low parameter errors and acceptable RMSE 0.027 and 0.029, respectively.

**Table 4:** Comparison of predicted and observed *μ*_max_ values as a part of internal cross-validation.

Comparison between	Linear regression and statistical parameters					
	Intercept	SE	Slope	SE	RMSE	R^2^
predicted LM-P and observed LM-S *μ*_max_ values	−0.0047	0.0093	1.0544	0.0181	0.0270	0.9927
predicted LM-S and observed LM-P *μ*_max_ values	0.0121	0.0097	0.9322	0.0176	0.0287	0.9912

The values predicted using the CM model for the LM-P strain slightly overestimated the experimental data for the LM-S strain. On the other hand, the calculated data for LM-S underestimated the experimental data for LM-P. However, the *B*_f_ values of 1.12 and 0.98 for the LM-P and LM-S CM models, respectively, were close to 1.0. Furthermore, Fig. 4 showed that, on average, the experimental values for LM-S were higher than for LM-P in the range of 6 °C–37 °C. However, the opposite situation could be seen in the temperature range between *T*_opt_ and *T*_max_ (Fig. 4).

Square root CMs for growth rate were externally validated using independent data from Combase at a_w_ of 0.974, pH 7 and from Bajard et al. [33]. For Combase data, the bias factors for the LM-P and LM-S models in SCM were 0.93 and 0.96, respectively, while the accuracy factors were 1.09 and 1.07, respectively. This means that the sqrtCM model for both *L. monocytogenes* strains provided a ‘fail-safe’ prediction to Combase data with 9 % and 7 % accuracy. On the other hand, the validation of LM-P and LM-S models based on data from this study in TSB with experimental growth rates by Bajard et al. [33] performed in Mueller-Hinton broth revealed ‘fail-dangerous’ predictions with *B*_f_ 1.06 and 1.07, respectively. Both accuracy factors were equal to 1.15.

## 4 Conclusions

In this work, the question of whether persistent or sporadically *L. monocytogenes* strains can grow better was dealt with by a quantitative study using two strains in two media in response to various temperatures. In the suboptimal temperature range, the growth parameters of persistent and sporadic, potentially non-persistent strains were almost indistinguishable, and it seems that growth predictions of persistent strains may be performed using the data of non-persistent strains (and vice versa), e.g., in exposure assessment. However, the models revealed differences in the behaviour of the strains at 40 °C and 43 °C. The results suggest that secondary modelling is suitable for testing a panel of *L. monocytogenes* strains regarding the possible correlation of their growth characteristics with persistence in food processing environments. The approach used in this study proved to be effective in revealing different growth responses in the range of higher temperatures than *T*_opt_. It can be extended also for mild inactivation temperature range as some similar differences in behaviour between persistent and non-persistent strains might also be expected.

## CRediT authorship contribution statement

**Ľubomír Valík:** Writing – review & editing, Writing – original draft, Visualization, Validation, Supervision, Software, Methodology, Funding acquisition, Conceptualization. **Jana Minarovičová:** Supervision, Methodology, Investigation. **Eva Kaclíková:** Writing – review & editing, Writing – original draft, Supervision, Methodology. **Adriana Véghová:** Methodology, Investigation. **Tomáš Kuchta:** Writing – review & editing, Writing – original draft, Resources, Conceptualization.

## Declaration of competing interest

The authors declare that they have no known competing financial interests or personal relationships that could have appeared to influence the work reported in this paper.
