# Supplementary material for: Modelling growth of two Listeria monocytogenes strains, persistent and non-persistent: Effect of temperature
Source: Heliyon. 2024 Dec 7;10(24):e40936. doi: 10.1016/j.heliyon.2024.e40936 (PMC11699068; doi:10.1016/j.heliyon.2024.e40936)
Supplement: Multimedia component 1 [file mmc1.docx]

Suppl. Table S1. Specific growth rates of the ComBase Predictor for *L. monocytogenes* that were used in the external validation of the rate models for the strains under study

| T (°C) | CBP *µ*_max_ | LM-P in SCM *µ*_max_ (*y*_cal_) | LM-S in SCM *µ*_max_ (*y*_cal_) | T (°C) | LM in MHB *µ*_max_ | LM-P in TSB *µ*_max_ (*y*_cal_) | LM-S in TSB µ_max_ (*y*_cal_) |
| --- | --- | --- | --- | --- | --- | --- | --- |
| 6  10  16  22  25  30  37  40 | 0.044  0.090  0.228  0.456  0.587  0.785  0.893  0.854 | 0.041  0.113  0.276  0.486  0.600  0.781  0.940  0.928 | 0.047  0.114  0.264  0.462  0.572  0.755  0.891  0.782 | 7.5  11  17  22  25  30  34  37 | 0.080  0.142  0.321  0.563  1.080  1.205  1.431  1.250 | 0.072  0.157  0.369  0.603  0.760  1.031  1.215  1.282 | 0.066  0.151  0.370  0.612  0.775  1.048  1.219  1.256 |
| CBP – ComBase Predictor; SCM - semi-synthetic cheese medium; MHB - Mueller-Hinton broth; TSB - Tryptic Soy Broth | | | | | | | |
